# Examining the Pathogenesis of Breast Cancer Using a Novel Agent-Based Model of Mammary Ductal Epithelium Dynamics

**DOI:** 10.1371/journal.pone.0064091

**Published:** 2013-05-21

**Authors:** Joaquin Chapa, Ryan J. Bourgo, Geoffrey L. Greene, Swati Kulkarni, Gary An

**Affiliations:** 1 Pritzker School of Medicine, University of Chicago, Chicago, Illinois, United States of America; 2 Ben May Department of Cancer Research, University of Chicago, Chicago, Illinois, United States of America; 3 Department of Surgery, University of Chicago, Chicago, Illinois, United States of America; University of South Alabama, United States of America

## Abstract

The study of the pathogenesis of breast cancer is challenged by the long time-course of the disease process and the multi-factorial nature of generating oncogenic insults. The characterization of the longitudinal pathogenesis of malignant transformation from baseline normal breast duct epithelial dynamics may provide vital insight into the cascading systems failure that leads to breast cancer. To this end, extensive information on the baseline behavior of normal mammary epithelium and breast cancer oncogenesis was integrated into a computational model termed the Ductal Epithelium Agent-Based Model (DEABM). The DEABM is composed of computational agents that behave according to rules established from published cellular and molecular mechanisms concerning breast duct epithelial dynamics and oncogenesis. The DEABM implements DNA damage and repair, cell division, genetic inheritance and simulates the local tissue environment with hormone excretion and receptor signaling. Unrepaired DNA damage impacts the integrity of the genome within individual cells, including a set of eight representative oncogenes and tumor suppressors previously implicated in breast cancer, with subsequent consequences on successive generations of cells. The DEABM reproduced cellular population dynamics seen during the menstrual cycle and pregnancy, and demonstrated the oncogenic effect of known genetic factors associated with breast cancer, namely *TP53* and *Myc*, in simulations spanning ∼40 years of simulated time. Simulations comparing normal to *BRCA1*-mutant breast tissue demonstrated rates of invasive cancer development similar to published epidemiologic data with respect to both cumulative incidence over time and estrogen-receptor status. Investigation of the modeling of ERα-positive (ER+) tumorigenesis led to a novel hypothesis implicating the transcription factor and tumor suppressor *RUNX3*. These data suggest that the DEABM can serve as a potentially valuable framework to augment the traditional investigatory workflow for future hypothesis generation and testing of the mechanisms of breast cancer oncogenesis.

## Introduction

The genesis and progression of breast cancer is a complex process involving multiple rare events that can occur over the lifetime of an individual [1]. Despite the continued effort to catalogue relevant genes, identify alterations in protein expression, and uncover novel pathways, the answers to many fundamental questions surrounding the mechanisms and complex interactions relevant to breast cancer oncogenesis remain elusive (reviewed in [2]). Attempting to characterize a thorough timeline and sequence of breast cancer pathogenesis presents several significant challenges. Firstly, the long and highly variable timespan over which oncogenic mutations accumulate to result in cancer makes longitudinal study of transformation nearly impossible. Secondly, because each genetic mutation is rare, the set of oncogenic events that produce any given cancer may be quite distinct, making it difficult to identify and contextualize the impact of individual events on normal cellular function. Lastly, it is becoming increasingly clear that while a number of significant pathways play a critical role in tumorigenesis (i.e. DNA damage repair, proliferation), the innumerable methods of pathway inactivation and molecular compensation result in a cellular environment too complex to decipher via the traditional reductionist paradigm of study [3]–[5]. Many of these challenges can be potentially met by the use of dynamic computational modeling to aid in the integration of existing mechanistic knowledge within a functional context that recapitulates a complex cellular environment [3]. As a result, we have developed a first-generation agent-based computational model to simulate the basic functional dynamics of the breast epithelium as related to normal physiology and the transition to breast cancer.

An agent-based model (ABM) consists of populations of computational entities (*agents*) that follow programmed rules governing their behavior with respect to the environment and interactions with other agents [6]–[9]. Often, known mechanisms of cellular behavior and responses are coded as conditional, *if-then* statements, and each type (or *class*) of agents is governed by its own set of such statements. Distinct rule sets simulate the diversity of responses to inputs and outputs that different cell types exhibit within a complex environment. The life cycle of each agent runs in parallel within a continuously interacting and changing environmental/cellular milieu, generating populations of agents mimicking the range of possible cellular behaviors of a particular type of cell. The dynamics of component interactions within the ABM can be observed throughout a simulation run, providing a degree of data resolution not possible in either experimental or clinical settings. This modeling framework was the basis of the work described below.

As greater than 95% of all breast malignancies are derived from epithelial lineage [10], examining the dynamic pathogenesis of breast tumors requires the ability to simulate the normal mechanistic dynamics of the mammary epithelium from which the cancers arise [11]. Toward this end, the Ductal Epithelium Agent-Based Model (DEABM) was developed such that the baseline dynamic state of the model, representing “health,” could give rise to aberrant conditions (i.e. cancer) by introducing recognized functional abnormalities into the cellular agent rule sets (e.g. a mutation inactivating a tumor suppressor). The construction of the baseline DEABM was based on published cellular and molecular mechanisms that govern the behavior of normal mammary epithelium. The complete details of model development and experimental procedures are presented in the *Materials & Methods* section.

The significant diversity among breast cancers challenges the ability to effectively capture and contextualize the dynamic nature of functional processes involved in the transformation of normal breast epithelium to malignancy. Attempts to provide order to this diversity include the use of a number of assays used to clinically classify breast cancers, such as OncotypeDx, PAM50 and Mammaprint [12], and molecular profiling studies, which have resulted in the recognition of distinct breast cancer subtypes [1], [13]. The striking finding from such studies is the heterogeneity of breast cancer, which greatly impacts biologic behavior and response to different therapies [14]–[16]. The identification of distinct breast cancer subtypes and their defining molecular features implies that breast cancers may develop via very different mechanisms. An effective model of breast tumorigenesis should be able to reproduce aspects of the diversity mentioned above. In attempt to simulate the functional molecular divergence of breast tumor types, development of the DEABM centered on representing the function of eight key oncogenes and tumor suppressors that play significant roles in both cellular function and breast cancer (Table 1). *While not intended to be a comprehensive catalog of breast oncogenes, the particular representative genes chosen were intended to illustrate key pathway dysfunctions leading to functional abnormalities observed in breast tumorigenesis.*

**Table 1:** Representative genes and their functions incorporated in the DEABM.

Gene Name	Function and Rationale for Inclusion	Role in Breast Cancer
***BRCA1***	Involved in DNA repair and G2/M progression. BRCA1 mutant families are more susceptible to breast cancer.	Suppressor
***RUNX3***	Modulates ER function and stability, Inhibits cMet expression and thus controls proliferative potential. May be associated with ER+ tumorigenesis.	Suppressor
***TP53***	Required for proper DNA damage response and repair. Tumor suppressor.	Suppressor.
***TGFBR3***	Loss of TGF- β receptor activity causes cells to be less responsive to inhibition of proliferation. Suppresses breast cancer progression.	Suppressor.
***CDH1***	E-cadherin mutations allow cell survival in the absence of cell-cell contacts. Frequently inactivated inbreast cancer.	Suppressor
***TERT***	Telomerase hyper-activation immortalizes cells beyond “Hayflick Limit.”	Oncogene
***MMP2/3***	Matrix-metalloprotease over-expression allows cells to breach basement membrane and promotes invasiveness	Oncogene
***MYC***	Strong oncogene associated with hyperproliferative phenotype in breast cancer.	Oncogene

Despite the ability to identify key lesions and subsequently categorize the behavior of individual breast tumors, there are still many unknowns concerning fundamental mechanisms behind the generation of the disparate sub-types of breast cancer [2]. A notable example is the pathogenesis of ER+ breast cancers. ER+ tumors make up the vast majority of breast cancers, but ER+ cells constitute a small percentage of normal breast ductal epithelium (between 4–12%) [17]–[22]. Furthermore, ER+ cells do not have proliferative potential, thus making it unclear how mutations to these cells could accumulate, be passed on to successive generations and progress to cancer. Correspondingly, since the DEABM is based on this existing knowledge, during initial development of the DEABM it was not possible to generate ER+ tumors. *This discrepancy between the current state of mechanistic knowledge (represented in the DEABM) and the recognized real-world preponderance of ER+ cancers led us to posit that a key functional gap in the current state of knowledge concerning breast tumorigenesis was in accounting for the control structure governing the proliferative potential of ER+ cells. This recognition led to a model-driven search for a putative mechanism by which ER+ luminal epithelial cells could be made to divide, which subsequently identified the function of runt-related transcription factor 3 (RUNX3) as having a potential role in the origin of ER+ tumors* [*23*]–[*25*]. The details of this process will be more comprehensively described in the *Materials & Methods*. Consequently, the incorporation of the function of RUNX3 into the DEABM allowed the simulation of ER+ tumorigenesis (see *Results* for more details). *RUNX3* functions as a transcription factor and has been shown to modulate the transcriptional activity and stability of ERα [25], [26]. The *RUNX3* gene is located on chromosome 1 at *1p36*, a locus that is frequently disrupted in breast cancer [25]. It has also been demonstrated as a tumor suppressor, whose disruption is considered an early event in breast oncogenesis [24], [25]. Interestingly, the vast majority of mutations that result in ER+ tumors in human models produce ER− tumors in mouse models; this is not true of *RUNX3* ^+/−^ mice, which spontaneously develop ER+ mammary tumors [25]. Additionally, nearly 50% of human breast cancers do not express *RUNX3* and loss of expression is associated with ER positivity [26]–[28]. Combined with the ability of *RUNX3* to impinge upon estrogen receptor function, these data suggest that *RUNX3* could play a potentially significant role in the development of ER+ breast cancer *in vivo*.

## Overview of the DEABM: Rationale and Representation

**Figure 1:**
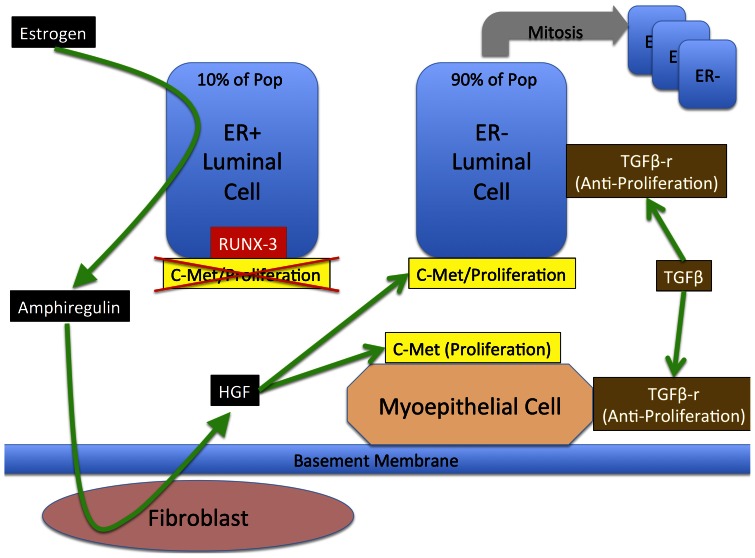
Overall schematic of cell-types and their interactions involved in duct epithelial cell life cycle. This figure depicts the minimally sufficient set of cell types and their interactions necessary to represent the growth and maintenance of the breast duct epithelial cell population. In particular note that given this representation ER+ cells do not have proliferative potential, a state that is maintained through the suppression of cMet by RUNX3.

**Figure 2:**
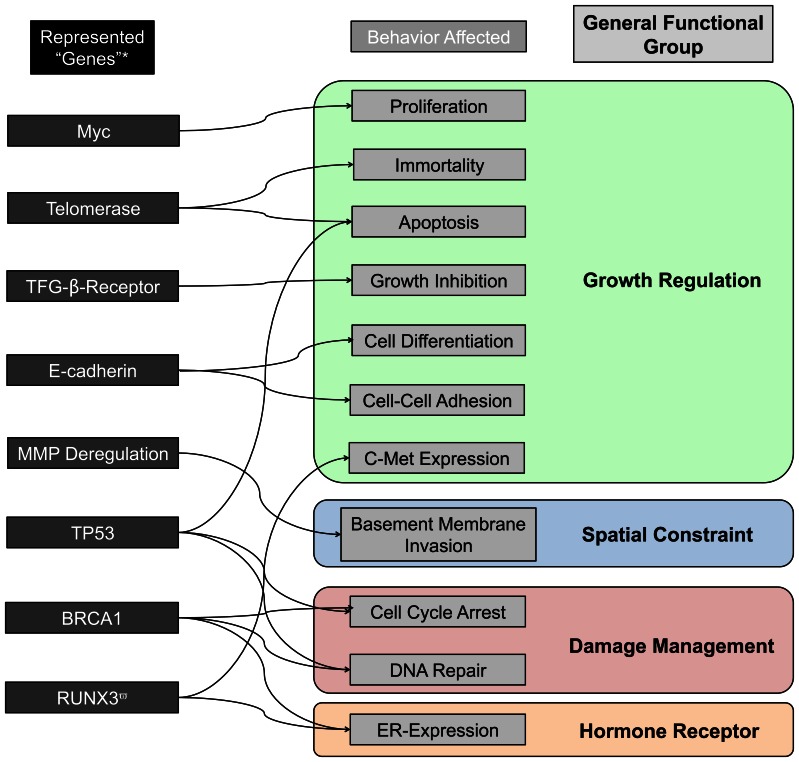
Set of included representative “genes” and their relationship to cellular behaviors and general functions within the DEABM. As the representational focus of the DEABM is on characterizing the functional dynamics associated with oncogenesis, potentially detrimental “genes” have been included on their known influences on those functions that are plausibly involved and altered in the process of tumorigenesis. Additionally, the arrows are intended to represent known direct regulatory effects; it is expected that there are many second and third order effects that might lead a named gene to affect other downstream behaviors. *Note that the labeling of these “genes” is not intended to be a comprehensive description of all the known effects of the named genes, but rather to label certain putative cellular behaviors possibly involved in malignant transformation.

**Figure 3:**
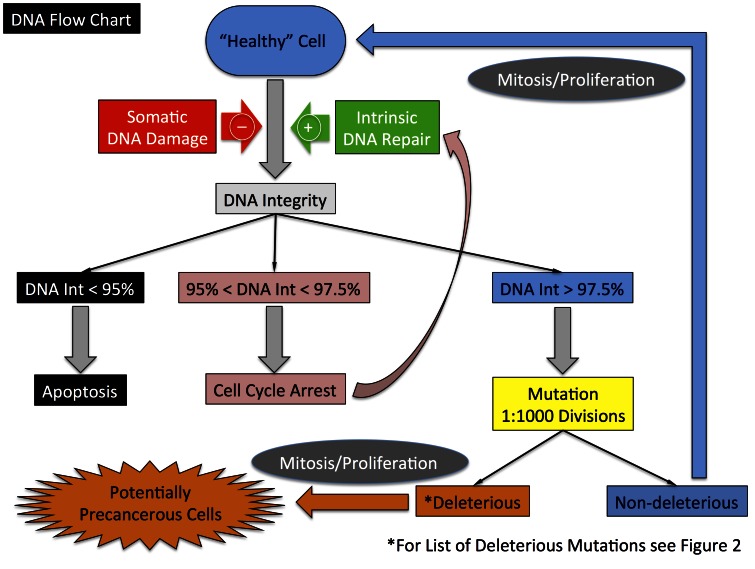
Schematic of control logic concerning DNA damage, repair and functional consequences of unrepaired DNA damage within the DEABM. A baseline premise of the DEABM is that DNA damage can occur during a luminal epithelial cell’s life-time, and that damage that remains unrepaired by the time the cell is to divide can be passed on as a mutation, a certain subset of which may affect a critical cellular function that may influence tumorigenesis. The DEABM incorporates abstract representations of DNA damage, damage repair, senescence, apoptosis and passage of mutations to subsequent cellular generations.

The modeling rationale behind the development of the DEABM is based on the concept of dynamic knowledge representation [29]–[31], with a focus on determining whether a particular hypothesis structure based on the current state of mechanistic knowledge is *sufficient* to explain a series of recognizable behaviors present in breast tissue. The iterative nature of this process is implicit, and involves the progressive addition of details only as existing models are deemed insufficient to reproduce selected behaviors in the targeted real-world systems [32]–[34]. Such an approach also follows the standard of successive tiers of validation present in the Modeling and Simulation community, specifically emphasizing the utility of the most basic and fundamental level of validation: *face validity*, which requires that a model must at least behave in a plausible fashion [35]–[37]. As such, by focusing on representing the dynamic consequences of known mechanisms and recognized functions manifesting in the behavior of cellular populations, as opposed to attempting to produce a high-resolution replication of breast histology, the DEABM abstractly represents breast tissue as a two-dimensional section of bilayered mammary ductal epithelium, envisioned as a portion of duct split open and laid flat. The cellular types represented as computational agents are those necessary for the maintenance of the duct epithelial population: luminal and myoepithelial cells, as well as their stem and progenitor populations, and fibroblasts. Figure 1 depicts the overall interactions between these cell types. Cell-level agents have internal state variables that represent molecular-level components of the cells: receptors, signaling molecules, genes, gene transcription factors, telomere length and DNA lesions (Table 2). Molecular pathways and molecular interactions were abstractly represented using logic-based and simple algebraic rules. Cellular agents execute their rule sets as the simulation iterates, thus interacting with other agents as well as the environment. Rules were organized into a series of functional modules, which could be affected by 8 focus gene-variables (Table 1). Seven of these genes-variables were selected as representative controllers for various functions that could be invoked in oncogenesis, such as loss of growth inhibition, loss of limits on cell divisions, impairment of DNA repair mechanisms, impairment of apoptosis and ability to invade the basement membrane (note some of these functions are replicated by the selected representative genes). The eighth, RUNX3, was chosen not for direct oncogenic potential, but for its role in preventing ER+ cells from dividing. For a list of these “genes” and their functional consequences see Table 1 and Figure 2; a more comprehensive description of the relationship between these genes-variables and the functional modules can be found in the *Material & Methods*. Cellular agents possess two functional copies of the listed “genes;” the presence of one copy was sufficient for the represented function to be maintained. As the cellular agents experienced DNA damage, if their DNA repair mechanisms were unable to completely clear this damage any gene copy mutations generated could be transmitted to subsequent generations of cells (see Figure 3). Mutations that resulted in the complete loss of function of one or a combination of the eight focus genes would lead to subsequent populations of cells of increased fragility and oncogenic potential. This model structure allows for the accumulation of mutations over time, thus reproducing the successive loss of serial functions within cellular populations as consistent with the biological reality. Global hormone levels (estrogen and progesterone) were applied based on known trajectories associated a 28-day cycle, representing the average human menstrual cycle, as well as pregnancy in subsequent calibration simulations, according to previously published reference values [36]. State variables are updated and agents execute the rules governing their behavior with each time step of the model; for the current DEABM, 1 time step is equivalent to 1 day. This timescale was chosen to reflect the length of the overall cell-cycle period observed in mammary epithelial cell division [37]–[39], and as such abstracts the intermediate states present during the cellular division process. More details of the implementation of the DEABM can be seen in the *Material & Methods*. The complete code of the DEABM can be downloaded from http://bionetgen.org/SCAI-wiki/index.php/Main_Page.

**Table 2:** Agent classes and their associated variables.

Agent Types	Variables	Description
**Turtles (Luminal** **cells, myoepithelial cells, progenitors, stem cells)**		
	Estrogen-Receptor	Recipient of Estrogen signaling, carries value = 1 if expressed by the cell agent, 0 if not.
	Progesterone-Receptor	Recipient of Progesterone signaling, carries value = 1 if expressed by the cell agent, 0 if not.
	c-Met expression	Expression of c-Met receptor, carries value = 1 if expressed of by cell agent, 0 if not.
	Hayflick-number	Telomere length, hayflick-number >40 ceases cell division
	bax-level	Pro-apoptotic protein, bax-level >60 induces apoptosis
	DNA-integrity	Accumulated DNA damage
	mutations	Accumulated somatic mutations
	Cell-cycle-arrest	Cell cycle arrest, set to 1 if dna-integrity<arrest-threshold
	tgfb-copies	Functioning TGF-beta genes (0, 1 or 2)
	p53-copies	Functioning p53 genes (0, 1 or 2)
	brca1-copies	Functioning BRCA genes (0, 1 or 2)
	e-cadherin copies	Functioning E-cadherin genes (0, 1 or 2)
	Myc-copies	Functioning c-Myc genes (0, 1 or 2)
	Telomerase-expressiom	Activated genes associated with telomerase expression (0, 1 or 2)
	MMP-expression	Activated genes associated with MMP secretion (0, 1 or 2)
	RUNX3-copies	Functioning RUNX3 genes (0, 1 or 2)
**Patch-environmental variables**		
	AREG-receptor	Receptor for Amphiregulin
	Amphiregulin-level	Local concentration of Amphiregulin
	HGF-level	Local concentration of HGF
	Rank-level	Local concentration of HGF
	TGFB-level	Local concentration of TGFB
	Basement-membrane	Value = 1 if a patch possesses basement-membrane, 0 if not

## Simulation Experiments

### Calibration and Validation of Breast Cell Population Dynamics in Normal Menstrual Cycles and Pregnancy

The initial simulations using the DEABM were focused on confirming the modeled breast cell dynamics behaved in a plausible fashion by corresponding to expected population trajectories in response to changing hormonal stimuli. This step is the necessary initial determination of *face validity* [35]–[37] of the DEABM. Values for estrogen and progesterone during the regular menstrual cycle [38], and pregnancy [39], [40] were based on previously published reference values. The curves for estrogen and progesterone fluctuations over a 28-day cycle were applied to the DEABM, and fluctuations in luminal cell population level were generated and assessed. The desired face-validation criteria included: 1) the maintenance of a dynamically steady state of the cellular populations, meaning that there was not either continually increasing size or progressive extinction of the population [41], and 2) a qualitative pattern matching of the population fluctuations to those expected from basic knowledge of breast physiology and extrapolated from proliferation metrics present in the literature [41]–[43]. Calibration to this behavior primarily involved tuning the parameters involved in cell division and cell death to obtain this dynamic steady state; the Supplementary Materials 1 provides a listing of the tuned parameters as well as an overview of the calibration process.

### Simulation of Longitudinal Patient Cohorts to Examine the Rate and Receptor Characteristics of Breast Tumorigensis

Following baseline calibration of the DEABM simulation experiments were performed to examine the time course of potential breast tumorigenesis from menarche to menopause. While we recognize that the majority of breast cancers occur in the post-menopausal period, we have focused the initial experiments using the DEABM on the pre-menopausal period for the following reasons:

1. Effectively characterizing the pathophysiology of any disease process is predicated upon characterizing the transition for the healthy/baseline state to the altered state. Since menopause follows decades of hormonal cycling, the state of the post-menopausal breast must be viewed as an alteration of the pre-menopausal breast. Therefore, oncogenesis in the post-menopausal breast can be most effectively evaluated when placed in context against the mechanisms of oncogenesis in the pre-menopausal breast (to be the subject of future investigations).
2. Known genetic predispositions for developing breast cancer, notably the effect of the *TP53 and BRCA1* mutations, primarily affects the pre-menopausal population. Therefore, in order to provide an additional comparison data set for the DEABM, focus is directed to the pre-menopausal period.

The initial simulated experiments were run for 15,000 steps (i.e. iterations during a single simulation run), representing a time period between menarche and menopause of approximately 40 years. Simulations were run in both the wild-type condition and a selected set of known oncogenic mutations: *TP53, Myc and BRCA1,* where single copies of each of these genes were altered at the initiation of each simulation run (n-individual simulations = 500 in each group, with N-groups = 3). We elected to carry out the simulation experiments in this fashion, with 3 simulated populations of 500 as opposed to one large population of 1500, to more effectively demonstrate how the DEABM could compared to existing published data sets. Outcome measures were the total number of runs that developed cancer by the onset of menopause, cumulative incidence rates by age and the proportion of cancers that were ER+, with ER expression in greater than 9% of cells defining ER+ status of a generated tumor. Cancer was denoted by expansion of the luminal cell population to greater than 10× the normal cellular population, a point demonstrated in preliminary simulations to eventually result in complete overgrowth of the model world, implying the presence of enough derangement of the system to correspond to unconstrained growth. As with prior calibration procedures, an initial set of parameters related to the different cell fates based on degree of DNA integrity were arbitrarily fixed at the levels seen in Figure 3 and the mutation rate adjusted to match the wild-type/sporadic cancer rate from the SEER review [44]. See the *Materials & Methods and Supplemental Materials S1* for a more detailed description of the model development and calibration process.

## Results

The genesis of breast cancer is a highly variable process that is poorly mechanistically understood [2]. The development of a complementary computational modeling system is a significant step that builds upon traditional reductionist approaches and facilitates the generation and initial evaluation of novel hypotheses aimed at probing the origins of breast cancer.

**Figure 4:**
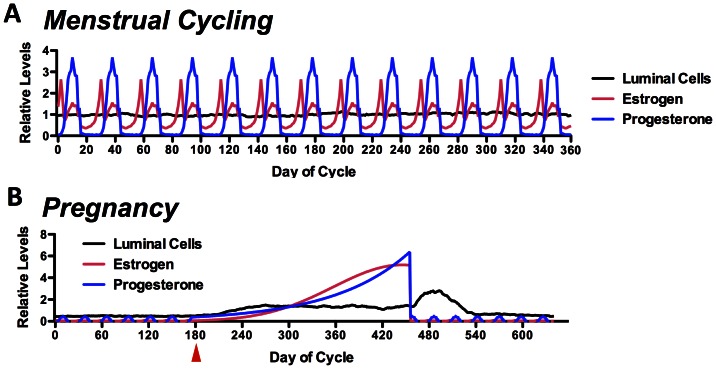
Post-calibration behavior of the DEABM reproducing baseline, normal breast epithelial dynamics. These graphs demonstrate the ability of the DEABM to generate recognizable fluctuations in luminal cell mass during normal menses (Letter A), demonstrating the first stage of the face validity of the DEABM in being able to reproduce self-sustaining cellular population without evidence of unconstrained growth. Furthermore, the DEABM was also able to reproduce expected alterations in luminal cell population dynamics associated with pregnancy, initiation depicted by red arrow (Letter B). These data.

**Figure 5:**
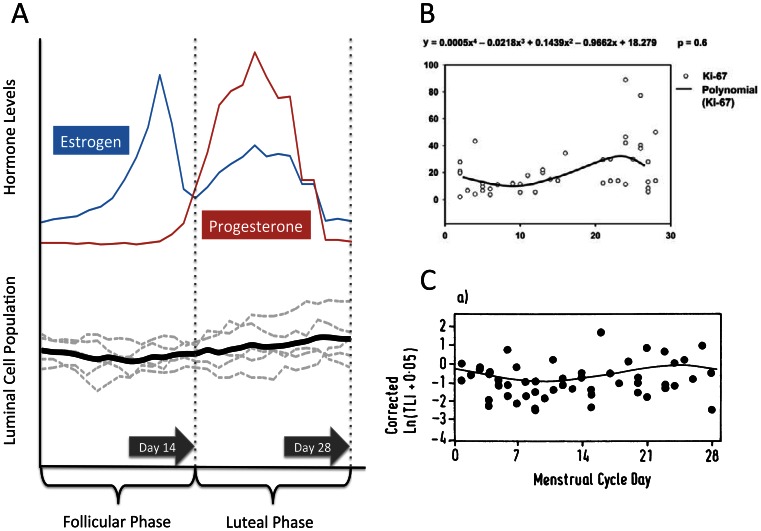
Demonstration of the DEABM to reproduce expected patterns of luminal cell growth in response to estrogen and progesterone within a menstrual cycle. Panel A depicts the output of the DEABM in terms of differentiated luminal cell population during the course of a single menstrual cycle period. The individual runs (n = 5) are depicted in light grey dashed plots and demonstrate the inter-run variance expected from the stochastic nature of the DEABM. The average of these runs is seen in the solid black line, and reproduces the expected increase in luminal cell mass seen during the luteal phase. The general trajectory of the DEABM seen in Panel A is noted to be similar to reference data sets present in the literature, as seen in Panel B (reproduced with under the Creative Commons License from Ref [42]) and Panel C (reproduced with permission from Ref [43]); both of which depict the degree of luminal cell proliferation during various phases of the menstrual cycle. Note in particular the wide variance in the sample points present in the reference data sets, which represent multiple samples obtained from multiple individuals.

Herein, a novel ABM was developed to integrate and instantiate a minimally sufficient conceptual model able to simulate the entire life cycle of breast epithelial cells, including the response to normal functions such as menstrual cycles and pregnancy. Normal menstrual-cycle response by the DEABM is illustrated in Figure 4A, which demonstrates the cycling hormone levels of both estrogen and progesterone over the course of a 1-year simulation and where the number of ductal luminal cells remains at a steady-state despite the physiological fluctuation of hormone levels during the menstrual cycle. This behavior meets the standard of face validity, insomuch that the cellular population does not exhibit continued growth or passage to extinction within the timeframe of the simulation. The DEABM’s simulated response of the breast to pregnancy is seen in Figure 4B, which demonstrated a 2.7-fold increase in luminal cell number sustained during pregnancy and decrease of cell population fluctuations, corresponding to the expansion of the mammary ductal structures in preparation for and during lactation [11], [41]. The next level of validation testing involved determining if the DEABM plausibly reproduced the general pattern of luminal cell population fluctuations during a menstrual cycle. Figure 5A depicts the results of 5 simulation runs and their average trajectory over the course of a single menstrual cycle, demonstrating a plausible result consistent with the known expansion of the luminal cell population during the luteal phase of the menstrual cycle. A comparison is made to two reference data sets (Figures 5B from Ref [42] and 5C from [43]). We believe that the represented outputs are functionally comparable and demonstrate plausibly correct behavior on the part of the DEABM (for a more detailed discussion concerning this comparison, see the *Discussion*). These experiments establish the face-validity of the DEABM in its ability faithfully model normal mammary physiology manifested as cell population dynamics.

The next step in the development of the DEABM involved evaluating its ability to generate realistic cancer incidences over an extended period of simulated time (∼40 years from menarche to menopause). It is worth noting that at this point historically during our development process the DEABM only included 7 functional types of genes (absent *RUNX3*). As evidence of the utility of the iterative process focused on establishing face validity, during the calibration process for mutation rates it became evident that the DEABM was behaving in a significantly implausible fashion. While its parameters could be tuned to generate realistic overall and longitudinal cancer incidences, the DEABM could not generate any ER+ tumors. As ER status is perhaps currently the most clinically significant defining tumor characteristic that guides breast tumor treatment, the face validity of the DEABM depended on its ability to generate a simulated tumor population with ER status comparable to its real-life counterpart. It was readily apparent that this was not an issue of parameter fitting, but rather represented an insufficiency of the underlying conceptual model. Specifically, the generation of cancer in the DEABM required mutated cells to divide, and in the DEABM, given its rules at that stage of development, ER+ cells could not divide. This led to the realization that, functionally, the genesis of ER+ cancers may require some means of reversing the suppression of the proliferation potential of ER+ luminal cells. Since, in the DEABM, suppression of ER+ proliferation was achieved by suppression of the receptor c-Met, a literature search was performed looking for a gene that performed this function. The result of this search suggested that perhaps *RUNX3*, which is capable of modulating ER transcriptional activity and stability, could also play a role in the generation of ER+ versus ER− breast cancers. It was at this point that *RUNX3* was incorporated into the DEABM. See the *Materials & Methods* for more details as to the rules associated with *RUNX3* and the means by which ER status on tumors were implemented.

Furthermore, during the course of performing the cancer incidence simulations it became apparent that the generation of cancers required a succession of functional failures. No single function loss alone was sufficient to generate a cancer; rather cancerous behavior required loss of both growth regulation (reflected by alterations to *Myc*, TGF-β-receptor, E-cadherin and telomerase) in combination with loss of spatial containment (reflected by the over expression of *MMP2/3*). Loss of DNA repair capacity (reflected in alterations to *TP53* and *BRCA1*) predisposed to the formation of mutated functions, whereas *RUNX3* affected ER status, as noted above. We wish to emphasize, however, that this finding does not imply that dysfunction of these specific genes are required for oncogenesis, but rather, in keeping with the functional emphasis of the DEABM, that there may be identifiable patterns of functional impairment that are associated with the generative history of breast cancers. We will elaborate on this subject in the *Discussion*.

**Figure 6:**
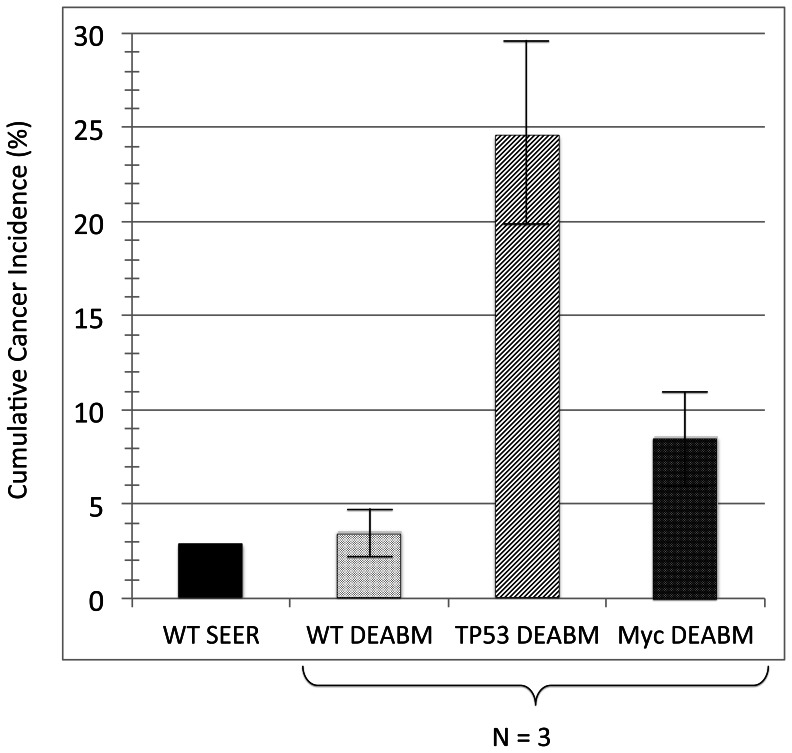
Comparison of cumulative cancer incidence generated in the DEABM for wild-type/sporadic, *TP53* and *Myc* mutation populations, as compared to the sporadic cumulative cancer incidence at age 55 reported in the SEER review. Cumulative cancer risk following ∼40 simulated years of menstrual cycles (15000 steps), n-individual simulations = 500 in each group (N-group = 3). The baseline sporadic cancer risk was ∼3.6 (range 2.3 to 4.9)%, similar to the cumulative incidence rate of 2.94% in 55 year-old women as reported in the SEER 2010 review [44]. Dysfunction of p53 resulted in a nearly 7-fold increase in cumulative risk of invasive breast cancer, 24.6 (range 19.8 to 29.4)%, and hyper-activity of the proto-oncogene *Myc* resulted in over 2-fold increase in cumulative risk to 8.6 (range 6.4 to 10.8)%. These data demonstrate the ability of the DEABM to generate recognizable and plausible increases in cumulative cancer risk associated with known oncogenic mutations.

The ability of the DEABM to effectively recapitulate the development and progression to malignancy corresponding to known genetic abnormalities associated with tumorigenesis was initially evaluated in simulations in which, a single copy of either *TP53* or *Myc* was “mutated” within the DEABM and experimental simulations (n-individual simulations per group = 500; N-groups = 3) were conducted to determine alterations in the frequency of the occurrence of invasive cancer. Simulations of the wild-type/sporadic breast cancer group (i.e. no pre-existing genetic abnormalities) demonstrated a 3.6 (range 2.3 to 4.9)% cumulative incidence of cancer at the end of ∼40 simulated years of menstrual cycles, not too dissimilar to the cumulative incidence rate of 2.94% in 55 year-old women as reported in the SEER 2010 review [44]. Compared to this group, the loss of function of p53 resulted in an expected, based on our knowledge of Li Fraumani Syndrome [45], nearly 7-fold increase in cumulative risk of invasive breast cancer, 24.6 (range 19.8 to 29.4)%, by the age of 55 (Figure 6). Similarly, the hyper-activity of the proto-oncogene *Myc* resulted in over 2-fold increase in cumulative risk to 8.6 (range 6.4 to 10.8)% under the same conditions (Figure 6).

**Figure 7:**
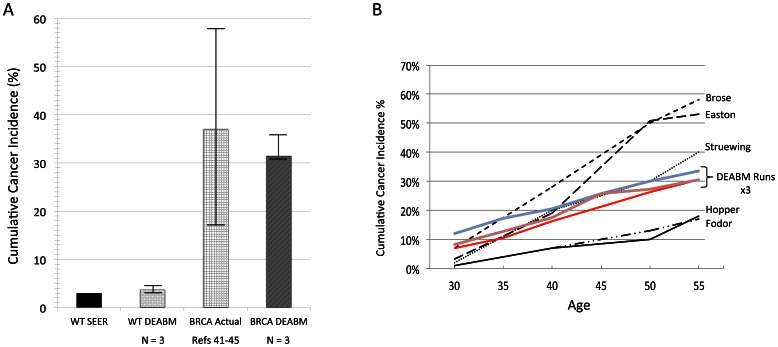
Demonstration of the ability of the DEABM to recapitulate cumulative and longitudinal cancer incidence in populations with BRCA1 mutations. Panel A demonstrates that the DEABM generated an increased aggregate cancer incidence over 40 years of menstrual cycles of ∼31.6% (range from 30.6–33.6%, N-groups = 3) compared to both sporadic/wild-type simulations and the SEER review data. The simulated BRCA1 values all fall well within the range of cumulative cancer incidences reported in BRCA1 population studies between 17–58% [46]–[50]. Panel B demonstrates that in addition to reproducing plausible cumulative cancer incidences, there is similar matching between longitudinal incidence over this ∼40 year interval between the output of DEABM simulations and the aggregated data from the previously noted studies on BRCA1 [46]–[50]. Published BRCA1 population study plots are labeled by study author name, whereas the individual DEABM cohorts of n-individuals = 500 are labeled by N-group number.

These results suggest that the DEABM was capable of simulating sporadic tumorigenesis at a single endpoint (overall cancer incidence), and recapitulating the known functional consequences of both tumor suppressors and oncogenes; subsequent simulation experiments were performed to determine whether it could reproduce a similar effect over an entire time course. Because women with a germline mutation in *BRCA1* have such a drastically increased incidence in breast cancer risk, this mutation afforded the opportunity to examine how the DEABM would perform in reproducing cumulative risk of breast cancer over time. As such, a single germline copy of BRCA1 was disabled in the DEABM to simulate a familial germline *BRCA1* mutation. Strikingly, the DEABM was able to reproduce the notable increase in breast cancer susceptibility seen in BRCA patients by 55 years of age (reference range of cumulative cancer incidence ranging from 17–58%) [46]–[50], with simulation results demonstrating cumulative cancer incidences increasing to 31.6% BRCA1 (range from 30.6–33.6%) from the 3.6% incidence seen the sporadic group (Figure 7A). In addition, the longitudinal cumulative incidence over time predicted by the DEABM was comparable to previously assessed risk in populations with germline *BRCA1* mutation [46]–[50] (Figure 7B), demonstrating the ability of the DEABM to plausibly reproduce the incidences of invasive breast cancer in both wild-type/sporadic and *BRCA1* mutant populations.

**Figure 8:**
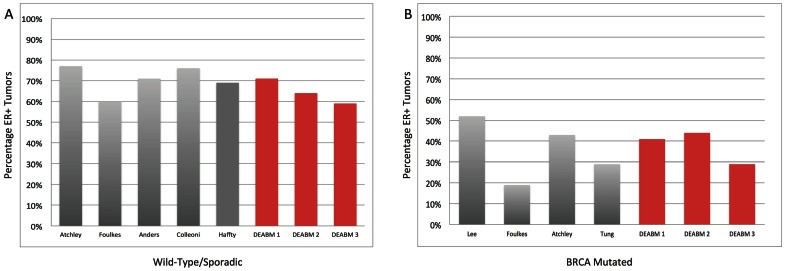
Reproduction of ER tumor status in both wild-type/sporadic and BRCA1 mutated populations of breast cancer. These data demonstrate the similarity between DEABM simulation runs and data extracted from the literature concerning the percentage of ER+ tumors generated in both wild-type/sporadic and BRCA1-mutated populations [46]–[57]. Panel A depicts the ER+ percentage among wild-type/sporadic populations from both the literature, ∼68% (range 60–77%) of premenopausal breast tumors [51]–[54], and in simulated populations (n-individuals = 500, N-groups = 3) of ∼65% (range 59–71%) of the simulated breast cancers. Panel B demonstrates the same comparison of ER+ tumors in the *BRCA1* mutant population, where the DEABM shows that only ∼38% (range 29–44%) of tumors generated were ER+ as compared to published incidences of ER+ *BRCA1* mutant tumors of ∼36% (range 19–52%) [51], [55]–[57]. For both Panel A and B published cancer population data is denoted by the name of the study’s first author, whereas the DEABM runs are labeled with their N-group number. These findings indicate that the DEABM incorporates plausible mechanisms for ER+ tumorigenesis, suggesting a role of *RUNX3* expression (or other genes performing a similar function) in the selectivity of ER+ breast cancer previously unknown.

**Figure 9:**
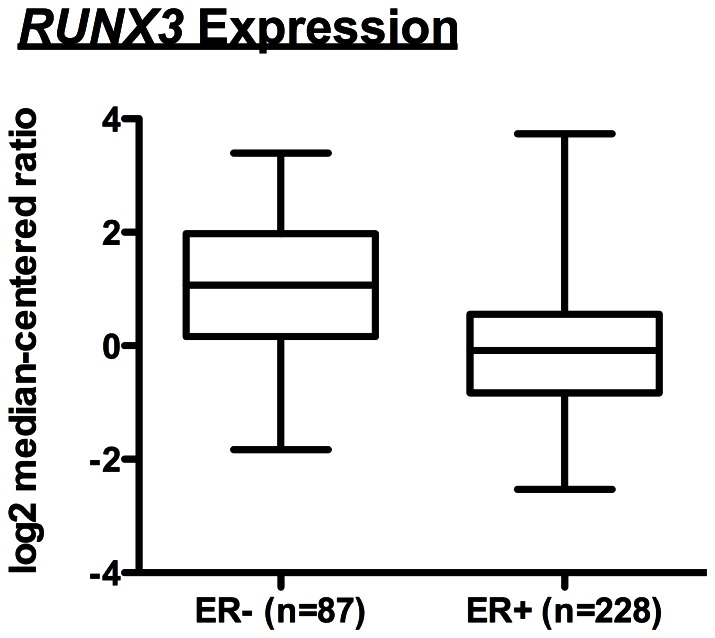
Relationship between RUNX3 expression and ER status in breast cancer. Graphical representation of search results from the Cancer Genome Atlas (TCGA) and Oncomine (www.oncomine.org) to identify correlations between RUNX3 expression and both ER+ and ER− breast tumors. These results suggest a trend towards decreased expression of RUNX3 in ER+ tumors, a finding consistent with our hypothesis that loss of RUNX3 function may be one of a class of genetic abnormalities that results in the loss of suppression of the proliferative potential of ER+ cells, and that this may be a precondition in the development of ER+ tumors.

Additionally, in terms of recapitulating ER status in these simulated populations, ∼65% (range 59–71%) of the simulated breast cancers in the wild-type/sporadic population were ER+ (Figure 8A). In comparison, a survey of the literature suggests that ∼68% (range 60–77%) of premenopausal breast tumors are ER+ [51]–[54]. These data suggest that the DEABM, following the inclusion of *RUNX3,* is plausibly simulating the key pathways that impact both breast tumorigenesis and hormone receptor maintenance. Similarly, in the *BRCA1* mutant population the DEABM shows that only ∼38% (range 29–44%) of tumors generated were ER+ (Figure 8B), in concordance to published incidences of ER+ *BRCA1* mutant tumors of ∼36% (range 19–52%) [51], [55]–[57]. These findings indicate that the DEABM incorporates plausible mechanisms for ER+ tumorigenesis, suggesting a role of *RUNX3* expression (or other genes performing a similar function) in the selectivity of ER+ breast cancer previously unknown. Encouragingly, post-simulation investigation identified data from The Cancer Genome Atlas (TCGA) and Oncomine (www.oncomine.org) that strongly suggest that downregulation or loss of *RUNX3* expression correlates to ER+ tumorigenesis (Figure 9). Combined with the findings that *RUNX3* is a modulator of ER function and stability, the DEABM assisted in implicating a role for *RUNX3* in the generation of ER+ versus ER− breast cancers. More importantly, these findings suggest a class of pre-pre-cancerous mutations that may set the stage for subsequent oncogene activation/tumor suppressor loss.

## Discussion

There is an increasing recognition that understanding the complexity of cellular systems will require methods of study that go beyond traditional reductionist approaches. Computational models, such as ABMs, have been proposed to play a vital role for the generation, and most importantly, testing of, hypotheses within this increasingly complex knowledge landscape [58]. Intrinsic to addressing the gulf between mechanistic knowledge and system level behavior is the recognition of the power of abstraction, with an emphasis on representing function and behavior. The application of the fundamental scientific principle that strives for generalization is crucial in attempts to place the high-dimensional data sets currently available into a mechanistic knowledge structure that will allow the engineering of therapeutic interventions. These concepts provide the rationale behind the development and use of the DEABM. As such, the DEABM is not intended to be a comprehensive depiction of the signaling pathways governing mammary epithelium. Rather, since the modeling goal is to examine cellular behavior at the system/population level (i.e. recognizing that tumors are populations of cells) in the transition from health to disease, the DEABM focuses on incorporating the primary mechanistic functions, cellular proliferation, apoptosis, paracrine signaling and genetic mutation, that are universally recognized as cellular processes. It is this emphasis on representing the interplay between well-defined, albeit abstractly represented, functional outputs in the transition for health to disease that sets the DEABM apart from prior computational modeling of the breast. Previous ABMs of breast cancer have focused on ductal carcinoma *in situ* (DCIS): a more theoretical cellular automata model of DCIS examines the effect of different proposed hierarchies of mammary stem on the generation of DCIS [59], an area of controversy explicitly not incorporated into the DEABM; whereas other projects examining the dynamics of the pathogenesis of specific subtypes of DCIS [60] and how pressure and mechanical forces might impact tumor microstructure [61], are aimed at examining particular sub-processes at a relatively high degree of mechanistic resolution and histological fidelity. Alternatively, the DEABM is intended to facilitate a more global examination of the *origins* of breast cancer, i.e the transition from healthy breast tissue to invasive cancer. The abstractions employed in the DEABM enhance the focus on this larger picture, demonstrating that abstract treatments of complex processes can be used to accurately represent global biology in both states of health and disease. This finding reinforces the implications of the nested nature of biological networks and the overall robustness of biological systems, with the attendant recognitions concerning the nature of system failure (i.e. multi-factorial and cascading) and the types of control needed to recover from failure [62]. Because the DEABM is the first attempt to model the endocrine and paracrine signaling networks that regulate the normal growth and involution dynamics of a mammary epithelial cell population, it was designed to be a minimally sufficient model to accomplish this goal. This approach is consistent with the modeling and simulation paradigm of progressive validation; as iterative model development progresses, additional detail can be included [31], [63]. Furthermore, it is important to note, both in ABMs generally and in the DEABM in particular, that the output data is not predetermined by the programmer, but arises from the complex interactions between agents. While some values were chosen arbitrarily because they “work”, this is not effectively different from standard theoretical models that describe complex systems without treating every detail with respect to enzyme kinetics, local concentrations and the full complexity of interactions present *in vivo*. To summarize, the DEABM was developed as a proof-of-concept first-generation modeling system that faithfully replicated a host of critical endpoints seen in the biology of breast tumorigenesis.

Despite the relative simplicity and abstraction of the DEABM, it was able to emulate a recognizable “normal” biological ductal epithelial life-cycle based on response to variations in growth factors and circulating hormones and incorporated the luminal cell population fluctuations and expansion that are seen with the menstrual cycle and pregnancy respectively. We acknowledge that the metrics produced by the DEABM do not exactly match the metrics present in the reference sets. However, these reference sets are themselves not without limitations and represent a coarse representation of the changes that occur to the breast epithelium during the menstrual cycle. The “snap shot” nature of pathologic evaluation cannot account for duration of events and rates of change; also, age, parity, length of the cycle and perimenopausal status can all cause significant variability in epithelial cell population response to circulating hormones [42], [43]. Nevertheless, we do believe that the represented outputs are functionally comparable and demonstrate plausibly correct behavior on the part of the DEABM. As we expand our model to include the aging breast (see below), many of these factors that can lead to significant variability will be incorporated. With respect to the generation of malignancy, the DEABM recapitulated cancer incidences in both sporadic cancer and *BRCA1* mutant simulations. Furthermore, the DEABM simulations of *BRCA1* mutant patients demonstrated a timeline of cumulative breast cancer risk comparable to that reported in the literature. This finding suggests that agent-based modeling is capable of dynamic behavior that accurately mimics the plasticity of human tissue over time. Future development of the DEABM will consist of adding more complex and detailed components and mechanisms with the goal of obtaining higher fidelity representation of the normal and neoplastic breast.

For example, the current DEABM has only been used to simulate pre-menopausal breast cancer, certainly a limitation of the model as most breast cancers occur in post-menopausal women. While there is almost certainly conservation of central processes between pre-menopausal and post-menopausal breast cancer, there is an equal certainty that there will be fundamental differences as well. Just as characterizing baseline “normal” breast epithelial dynamics can provide insight into the fundamental process structure of pre-menopausal tumorigenesis, we assert that simulating the local conditions (paracrine, autocrine and intracrine hormonal concentrations) that occur in the breast during the “normal” transition to menopause during the perimenopasual period will be a necessary step in characterizing the pathogenesis of post-menopausal breast cancer. Additionally, there is a growing recognition of the influence of other breast tissue components, such as adipose tissue and inflammatory cells, on the malignant transformation of breast epithelial cells. So whereas the initial build of the DEABM started with the most basic components necessary to reproduce baseline duct epithelial dynamics, we recognize the importance of representing and investigating how genetic alterations, hormones, and growth factors interact across different cellular and tissue components. Therefore, future development of the DEABM will involve the addition of these components and breast architectural units such as Terminal Ductal Lobular Unit, facilitating the extension of the DEABM to a more comprehensive Breast Tissue ABM.

Because the goal of the present research was to start with the basic dichotomy of ER+ and ER− tumors, representation of HER-2 overexpression was excluded. We recognize that for any sufficiently complex model that would probe the effects of breast cancer treatment (which the current version of the DEABM is not intended to do), HER-2 overexpression and associated mechanisms would be a necessary inclusion. At the current level of model resolution an initial goal of being able to represent the mammary epithelial life cycle HER-2 is not a *necessary* component, but the knowledge that HER-2 does play a clinically significant role would point future development of the DEABM in this direction. For instance, since HER-2 is known to be a member of the epidermal growth factor receptor (EGFR) protein family, and overexpression is seen in ∼20% of breast cancers [64], it can be readily seen by examining the DEABM rule set that there may be a cross-receptor activity between EGFR and HER-2 in breast fibroblasts in response to amphiregulin produced by estrogen-stimulated ER+ luminal cells, which in turn would increase the production of HGF to further stimulate proliferation. This is an example of the inevitable need to expand the range of representation included in the DEABM (and its successors), but also provides an example of how future development should progress in a systematic fashion. There is a temptation to try and “put it all in” and create as comprehensive a model as possible from the outset, based on the belief that understanding necessarily arises from volume of detail. While this approach can provide useful information and insight, we suggest that there is a complementary role for modeling projects that come from the other direction, focusing on building upon minimally sufficient abstractions that generate recognizable behaviors that can be subjected to successive tiers of validation. With this type of approach, additional detail is added when needed to represent a behavior or phenomenon that cannot be generated with the existing model. To this end, more molecular detail would be incorporated into the DEABM to recapitulate the histories associated with known molecular subtypes of breast cancer and further delineate as-yet-unknown intrinsic breast cancer subtypes that may occur with distinct patterns and temporal sequences of DNA damage, with the ultimate goal of accounting for intra-tumoral variation.

It is well accepted that the compounding of significant DNA mutation over the lifetime of an individual is the basis for tumorigenesis; this is consistent with the concept of cascading systems failure seen in multi-hierarchical systems [62]. However, the ability to faithfully track the impact of these mutations over the lifetime of a patient is not feasible. As such, the disparate sets of mutations, or any sequential dependencies, that may result in the genesis of ER+ versus ER− tumors remain elusive. However, this is a process that can be examined with agent-based modeling, as we have done with the inclusion of RUNX3 into the DEABM. After inclusion of the tumor suppressor *RUNX3*, the DEABM replicated the rate of ER-receptor positive tumor incidence with striking accuracy. However, RUNX3 was not added into the DEABM based on its known tumor suppressor capability, rather it was chosen for its effect on suppressing the proliferative potential of ER+ cells, a functional capability that needed to be added to the earlier version of the DEABM. As a result, in the DEABM the loss of *RUNX3* expression leads to an expanded ER+ population by allowing ER+ luminal cells to express c-Met and to receive proliferative stimulus via associated HGF signaling from nearby fibroblasts, i.e. allowing ER+ cells to exhibit the key behavior, replication, necessary for their subsequent mutations to manifest in successive generations. Interestingly, loss of TGF-β signaling, the upstream activator of *RUNX3*, produced the same effect. It has been convincingly demonstrated that RUNX3 acts as a tumor suppressor in breast cancer, and that it modulates the function of ERα [23], [25], [26]. *RUNX3* expression is also significantly lower in ER+ mammary ductal carcinomas (versus ER− cancers, see Figure 8), suggesting that dysregulation of *RUNX3* does play a role in the preference for ER+ breast tumorigenesis. The ABM provides a unique opportunity for us view some of our clinical categorizations from a different perspective. For example, in our model we chose a 9% ER positivity as our threshold for ER positive tumors, which is very close to the 10% quoted in the literature. This threshold was chosen because the percentage of ER positive cells in benign epithelium is approximately 9%. Some authors advocate a threshold of 1%, however, a lower threshold for ER positivity would necessitate down regulation of ER positive cells in our model that may be consistent with a partial loss of RUNX3 function or alternative mechanism that may represent a biologically different tumor. Further development of the model to identify the alterations found in tumors with low ER expression may help clarify the clinical threshold for treatment of ER positive disease. A future step for our current model could be to mimic current therapies for ER+ tumors that reduce the amount of available estrogen through receptor blockade or decreased availability. In addition to demonstrating known “therapeutic effects,” additional effects might be gleaned, or mechanisms of resistance may emerge. Other well studied targeted therapies such as trastuzumab or prospective treatments could be evaluated in a similar fashion.

The generation of largely ER− tumors with *BRCA1* mutant DEABM simulations further supports our hypothesis. It is well known that the overwhelming majority of people with germline *BRCA1* mutations develop basal-like (triple-negative) breast cancer. After the inclusion of *RUNX3* functionality, the DEABM generated nearly 60% of tumors as ER-, compared favorably to nearly 70% as reported in the literature. The ability of the model to mimic clinical rates of tumor ER-status so closely, particularly with regard to a mutation in a single gene (*BRCA1*) demonstrates the potential benefit and utility of model-aided hypothesis generation and evaluation. It is important to emphasize that the hypothesis on the role of *RUNX3* does not imply that it is the sole and unique mechanism for the genesis of ER+ tumors. While *RUNX3* was chosen for inclusion in the DEABM because of its multiple interaction points with pathways governing normal growth and inhibitory signaling networks, there are likely multiple potential control points involved in this process. For instance, the enrichment for non-silent *TP53* mutations in ER− breast cancer subtypes may potentially play a significant role in the selected generation of ER− cancers. Because both p53 and BRCA1 are involved in DNA damage response, it is likely that increasing sustained DNA damage is a risk factor for preferentially developing ER− breast cancer. In fact, evidence for this exists already, as the majority of ER− tumors are p53 positive which is indicative of the presence of the mutated protein [1].

Looking forward, the DEABM, and associated models, could be invaluable tools for testing novel hypotheses of oncogenesis based on the enormous amount of genomic information that is now available through programs such as The Cancer Genome Project. The clinical utility of much of this information is yet to be discovered, as doing so presents challenges in contextualizing such information into mechanistically plausible sequences of events within the (clearly recognized) protean natural histories of tumorigenesis. For instance, we suggest that the investigatory process depicted concerning RUNX3 could provide a guide for the adoption of an integrated computational-experimental workflow. The formal process of model-making forces explicit expression of what is known and understood about the system under study. The instantiation of that knowledge with the DEABM helps determine the sufficiency of that knowledge to explain known observations: in our case this was the lack of ER+ tumors. The explicit mechanistic representation within the DEABM pointed to a potential gap: the identification of c-Met as a control point for ER+ proliferation. This recognition led to a literature search that identified *RUNX3*, which when inserted into the DEABM, improved its ability match expected behaviors. The plausibility of the potential role of *RUNX3* was then further confirmed by the findings of the retroactive search of The Cancer Genome Atlas (TCGA) and Oncomine (www.oncomine.org) (Figure 8).

We assert that agent-based modeling could assist in this contextualization, by enhancing the investigation, understanding and categorization the multiple possible trajectories resulting from multiple sets of possible mutations into groups or classes of functional tumor phenotypes, with prognostic and therapeutic implications. Doing so would aid in the development of a functional “taxonomy” of breast tumors, aiding the ongoing process of molecular profiling by adding a behavioral layer of organization into which specific gene signatures could be placed. In summary, ABMs allow the visualization and measurement of complex systems over long periods of time in unlimited populations in a way that is not possible in either *in vitro* or *in vivo* systems. The innovation of such models is to provide a platform in which oncogenesis can be tracked across a series of identified mutations that progress from precancerous states to, ultimately, invasive cancer. Dynamic computational knowledge representation of this type introduces the ability to generate dynamic, functional maps of oncogenesis, and offers the promise of an additional means of better categorizing breast cancers.

## Materials and Methods

The *Materials & Methods* section is divided into a description of the development of the DEABM followed by a description of the simulation experiments carried out using the DEABM. The development of the DEABM followed the general process described in the *Overview, Design Concepts, Details (ODD)* protocol developed by Grimm, et al. [65], as modified to meet the specific needs of agent-based modeling of biomedical systems [5], [29]–[31], [66]–[69]. Additionally, the iterative nature of model development follows the process described in a series of studies that emphasize the successive addition of model features to match an increasing number of desired observables as a means of enhancing the scope of model representation [32]–[34]. The DEABM was implemented using NetLogo 5.0, which can be obtained online at http://ccl.northwestern.edu/netlogo/ [9]. Description of the simulation experiments include those intended to first test the validity of the DEABM in terms of effectively reproducing normal breast cell population dynamics in response to different normal hormone patterns (cyclical menses and pregnancy) without introducing mutations, then introducing mutations in order to validate the ability of the DEABM to reproduce recognized incidences of breast cancer development.

### Overview of DEABM Architecture

The DEABM represents a two-dimensional patch of bilayered mammary ductal epithelium, envisioned as a portion of duct split open and laid flat, as a two-dimensional, toroidal square grid populated by motile (luminal and myoepithelial cells, as well as their stem and progenitor populations) and immotile (fibroblasts) agents. See Figure 1 for the interactions between these cell types. In addition to providing locations for agents, grid spaces (“patches” in NetLogo terminology) also possess variables representing extracellular concentrations of hormones, mediators and the basement membrane. While the DEABM incorporates spatial effects, it does so primarily to allow representation of the dynamic consequences of spatial effects such as cellular crowding, cell mass expansion, and the locality of paracrine effects, in a relational fashion as opposed to a tissue realistic one. Thus the “space” of the DEABM is better viewed as a representation of the cellular interaction/communication network rather than as an attempt to replicate histological detail; this is in contrast to other ABMs of breast cancer, the work of Macklin [60] and Norton [61], which attempt to replicate specific morphological features of DCIS. Given the emphasis on the relational structure between the cellular populations, we utilize a ratio of 3 luminal cells to every 2 myoepithelial cells (based on extrapolating the findings from Van Keymeulen *et al*. [70]), abstracting the layered relationship between myoepithelial and luminal cells as the “carrying capacity” of each grid space. This allows the DEABM to represent some degree of spatial plausibility and constraint while representing the interactions and subsequent behaviors of the cellular populations as they accumulate mutations and start to exhibit behaviors that shift them from the healthy dynamic steady state. Table 2. lists the agent-types and their internal state variables. Molecular pathways and molecular interactions were abstractly represented using logic-based and simple algebraic rules. State variables are updated and agents execute the rules governing their behavior with each time step of the model; for the current DEABM, 1 time step is equivalent to 1 day. This timescale was specifically chosen to reflect the length of the cell-cycle period observed in mammary epithelial cell division [71]–[73]. Estrogen and progesterone levels are represented as global variables with levels set based on published values for a 28-day cycle at baseline, then altered for pregnancy [38]–[40], [74]. For a complete list of agent types and their respective variables see Table 2. The complete code of the model can be found online upon publication.

### Description of Secreted Mediators Implemented as Environmental Variables

Much of cell-to-cell communication takes place through the secretion and receptor-activation of mediators secreted into the extracellular space. The DEABM simulates this paracrine type of behavior through the use of environmental (“Patch”) variables, specifically representing amphiregulin, hepatocyte growth factor (HGF), RANK-ligand and transforming growth factor beta (TGF-β). These variables are listed in Table 2. The variables can be conceptually viewed as representing the amount of these signaling molecules present on a particular grid space. They are “produced” by the cellular agents based on specific rules (see below), and “absorbed” via receptor binding via a different set of rules (see below); these actions are manifest by the cellular agents either increasing or decreasing the value of the particular environmental variable on the grid space occupied by the cellular agent. Similarly, the value of the particular environmental variable on a grid space represents the strength of the signal sensed and transduced by the cellular agents residing on that grid space. To simulate the spread of these mediators through the extracellular milieu, the DEABM utilizes NetLogo’s “Diffuse” function, which takes a set percentage of the environmental variable’s value on a single grid space, reduces the value on that grid space by this amount and spreads the reducing amount into the surrounding grid spaces. As with all the parameters present in the DEABM, the diffusion rates are qualitatively calibrated in relation to each other, the behavior of the agents are checked against the global behavior of the system, and their values are listed in the Supplementary Materials 1.

The exception to the environmental/”Patch” variables noted above is the “Basement Membrane.” This variable is also a patch variable, meaning that it is not specifically intended to represent a descriptor of cellular state, but is not a diffusible entity. Rather, it is a property of the particular grid space, produced by and affecting the various cellular agents present on the particular grid space. For simplicity’s sake the Basement Membrane is represented as a binary variable: either it is present or not.

### Description of Cell Types Implemented as Agents

Since the primary focus of the DEABM is on the dynamics of ductal breast cancer oncogenesis, emphasis is placed on representing the components and factors potentially involved with respect to the life-cycle of the luminal epithelial cells. The other cellular types involved in the maintenance of the breast duct are represented with an emphasis on their role on luminal cell dynamics, and do not include their own capacity for malignant transformation. The interactions between the different cell types can be seen in Figure 1. The sections below describe the various characteristics of the cell-types included in the DEABM.

### Fibroblasts

Fibroblast function in the DEABM is heavily abstracted and is focused on their role in terms of the duct epithelial life cycle, with specific emphasis on their production of hepatocyte growth factor (HGF). They are non-motile and their own life cycle is not modeled in the DEABM. Their rules include:

1. Production of HGF when stimulated by amphiregulin, a ligand of epidermal growth factor receptor, which is produced by estrogen stimulated estrogen receptor positive (ER+) luminal cells [75].
2. The production of HGF is down regulated by Transforming Growth Factor-beta (TGF-β) [76]–[79].
3. HGF is the terminal mitogen that binds to the receptor c-Met on breast myoepithelial and luminal cells and induces proliferation [75].

### Myoepithelial Cells

Myoepithelial cells are attached to the basement membrane and form part of the structure of a breast duct that underlies the luminal epithelial cells [80], [81]. Their primary role in the DEABM is to generate and maintain the basement membrane, which provides a constraint on the growth dynamics of the luminal cells [81]. The life cycle of myoepithelial cells is also represented, including their respective stem and progenitor cells. In general, modeled myoepithelial cells do not undergo mutations, with the exception of an affect on their apoptosis function (see below).

#### Myoepithelial stem cells

As with the luminal stem cells (see below) the life cycle and internal details of myoepithelial stem cells is not modeled; in the DEABM they primarily serve to be the original source of myoepithelial cells. At initialization, a simulation run of the DEABM begins with 4 myoepithelial stem cells at the center of the world. The initialization period ends when the generated differentiated cell populations reach a dynamic steady state.

1. Division Rules: They divide based on the presence of HGF to produce one myoepithelial progenitor cell and one myoepithelial stem cell. As a result, there is never an expansion of the myoepithelial stem cell population itself.
2. Mutations: They do not accumulate damage or undergo mutations (in the DEABM).
3. Basement Membrane: They do produce and maintain the basement membrane on their resident patches (see below)

### Myoepithelial Progenitor Cells

1. Origins: Myoepithelial progenitor cells can be generated by one of 3 parent cell types, all in response to the presence of the proliferative signal RANK-ligand: 1) myoepithelial stem cells, 2) prior myoepithelial progenitor cells, or 3) differentiated myoepithelial cells (this last mechanism simulates mesenchymal transformation, see below). RANK-ligand is produced by stimulated progesterone receptor positive (PR+) luminal cells IF local (i.e. residing patch) HGF levels are sufficient AND the concentration of TGF-beta is below the inhibitory threshold [76]–[79].) The HGF effect occurs via its binding to the c-Met receptor on the myoepithelial progenitor cell.
2. Spatial Effects/Positioning: Myoepithelial progenitor cells move one grid-space/day in a random direction until they reach a patch that has a basement membrane AND is adjacent to at least one differentiated luminal cell AND at least one differentiated myoepithelial cell, at which point they cease movement and begin to differentiate. This simulates the effect of both binding to the basement membrane and cell-cell interactions mediated by E-cadherin, which must be expressed in order for this effect to occur [82]–[85].
3. Production of TGF-β: Myoepithelial progenitor cells secrete TGF- β in response to HGF [76]–[79].
4. Apoptosis: Myoepithelial progenitor cells that do not adhere and differentiate within 5 days will undergo apoptosis [86]–[88].
5. Mutations: Mutations of E-cadherin delay the initiation of apoptosis due to failure of adherence [82]–[85] (see corresponding section below in Luminal Cells for more details).

### Differentiated Myoepithelial Cells

1. Mesenchymal Transformation: Can undergo mesenchymal transformation and manifest proliferative potential by converting back to proliferative myoepithelial cells via activation of RANK receptors by RANK ligands produced by progesterone stimulated PR+ luminal cells [80].
2. Basement Membrane: Myoepithelial cells produce the basement membrane on its patch (represented as a binary patch variable denoting whether the basement member is present or not), and stimulate adjacent myoepithelial cells to produce the basement membrane on their respective patches [81]. If the myoepithelial cell dies (see below) then the basement membrane on the now unoccupied patch degrades over time.
3. Apoptosis: Myoepithelial cells undergo apoptosis. They have a state variable representing the apoptosis promoter Bax-1, which increases by 1 each day. Apoptosis occurs when the Bax-1 level reaches 60, consistent with the 60-day lifespan of mammary epithelial cells found in the literature [72], [89].
4. Production of TGF-β: Differentiated myoepithelial cells secrete TGF- β in response to HGF [76]–[79].

#### Luminal cells

Luminal (epithelial) cells have the highest degree of internal complexity of all the cell types represented in the DEABM. Specifically, they include rules for DNA damage/repair, cell cycle arrest, apoptosis, and functional mutations, as well as existing in two distinct populations of ER+ or ER− cells. They arise from stem cells through to proliferative luminal cells, but can also be stimulated to proliferative themselves if ER-. Given their complexity, their various functions are described in separate sections below:

#### Luminal stem cells

The DEABM does not attempt to describe regulation of stem cell proliferation and treats continual renewal of their own population as given. Initialization of each simulation run of the DEABM starts with 4 luminal stem cells in the center of the model world and is followed by an initialization period during which these stem cells generate sufficient luminal cells to reach a dynamic steady state.

1. Division: Luminal stem cells divide based on a stochastic process with a probability/day of 25% in the presence of HGF.
2. Outcome of mitosis: The mitotic products of luminal stem cell division are asymmetric. Mitosis results in self-renewal of the stem cell and the birth of one new progenitor cell with a genome identical to its parent stem cell. As a result, there is never an expansion of the luminal stem cell population itself.
3. Somatic functions: Luminal stem cells undergo DNA damage/repair, cell cycle arrest and mutations in the same manner as somatic luminal cells (see below).

#### Luminal progenitor cells

These cells represent the motile form of luminal cells immediately after division and before adherence.

1. Origins: Luminal progenitor cells are produced by both stem and differentiated luminal cells in response to HGF as long as NOT suppressed by TGF-β [76]–[79] AND they express the c-Met receptor to bind the HGF.
2. Secretion of TGF- β: They secrete TGF- β in response to HGF [76]–[79].
3. Spatial Effects/Positioning: This set of rules/conditions is intended to replicate a relatively basic behavior: the movement of luminal progenitor cells from where they arise (as daughter cells) to where they end up (as adherent differentiated luminal cells). Capturing this behavior in a dynamic computational model, however, requires a series of constraining attributes: 1) under normal circumstances these luminal cells do not grow in piles, i.e. the presence of a luminal cell in an area precludes a migrating progenitor luminal cell from occupying that exact same space, 2) there is a positional relationship between luminal cells and myoepithelial cells, i.e. the luminal cells next to the lumen and the myoepithelial cells adjacent to the basement membrane, and 3) the relative continuous/contiguous nature of the luminal epithelial layer. Therefore, in order for a luminal progenitor cell to find a place where it can adhere and differentiate requires it moving until the following conditions are met:
  - The grid space it moves on cannot have more than the allowable number of differentiated luminal cells (set as 3) already on it.
  - The grid space it moves on must have at least one myoepithelial cell with an intact basement membrane on the grid space.
  - The grid space it moves on must have at least one differentiated luminal cell directly on a directly adjacent grid space.

If these conditions are not met by the end of 5 days simulated time, the luminal progenitor cell will undergo apoptosis (see #4 immediately below). This set of rules enforces the spatial configuration and associated limits on growth patterns for the baseline DEABM in a fashion that we believe is consistent with its degree of spatial representation (i.e. no 3^rd^ Dimension or specific representation of histological tissue architecture) and reflects the role of myoepithelial cells in constraining luminal cell expansion [81].

4. Apoptosis: Luminal progenitor cells that do not adhere and differentiate within 5 days will undergo apoptosis, a process that is implicated in lumen formation and involution in normal menstrual cycles and the post-partum period [86]–[88].

5. Expression of TGF- β: They express TGF-β-receptor, which if bound to TGF-β slows their proliferation [76]–[79].

#### Differentiated luminal cells

Differentiated luminal cells do not change their position, representing their adherence to their underlying myoepithelial cells. They also secrete TGF- β in response to HGF [76]–[79]. Since the focus of the DEABM is on the potential for neoplastic transformation of this particular cell line, the internal functions of the luminal epithelial cells are modeled with a greater degree of detail than the other cell types, as described below:

### “Genes” as Functional Modules

The rule-based nature of the DEABM allows for a functional modular organization of its code, which can then be mapped to corresponding gene functions. A schematic listing modeled genes and their respective influences on luminal cell function can be seen in Figure 2. It should be noted that the list of modeled genes is not intended to be comprehensive, as it is clearly recognized that there are numerous genes involved in the cellular functions modeled; additionally, we certainly recognize that many of these genes have pleomorphic effects. Rather, since the goal at this stage of DEABM development is to demonstrate the capability of this type of model to reproduce recognizable dynamics, we have chosen to focus on a set of highly-studied representative genes. Each cell begins with two fully functional copies of eight genes that reflect known or hypothesized tumor suppressors or oncogenes. Acquired mutations to each gene contribute to oncogenesis, but none are sufficient for oncogenesis singularly. These genes/functional modules are then the targets for the mutations that can potentially occur due to DNA damage (see below).

1. Telomerase: Luminal cells can senesce based on telomerase function tied to their hayflick number, which is inherited from parent cells. This variable is increased by one following each round of cell division; upon reaching 40, cells are no longer able to divide further (i.e. enter senescence), simulating the known phenomenon of the limited replicative potential of cell lineages due to telomere shortening [90]. Failure of telomerase activation increases the number of divisions a cell line can propagate, potentially leading to immortalized lines.
2. E-cahedrin: As noted above, cell adherence and consequent initiation of differentiation is mediated by E-cadherin. E-cadherin is essential for luminal cell-cell adhesion, cellular differentiation, and resistance to apoptosis. Failure of E-cadherin delays the initiation of apoptosis in non-adhered cells [82]–[85], [91], [92].
3. TGF-β-receptor: Binding of TGF-β to the cell’s TGF-β-receptor slows proliferation. TGF-β-receptor activation is also necessary for the expression of RUNX3 (see below). Loss of TGF-β-receptor leads to reduced growth inhibition [76]–[79].
4. p53: p53 is involved in promoting the functions for DNA repair, entry into cell cycle arrest and the initiation of apoptosis (see below). It is complementary to the function of BRCA1 (see below), including its effect on ER expression [93]–[95]. Failure of p53 leads to impairment of all its associated functions.
5. Myc: Myc is a proto-oncogene that, if expressed, increases the likelihood of cell division. At baseline, Myc is suppressed, and mutations lead to loss of its suppression [96], [97].
6. Regulation of matrix metalloproteinases (MMP): Under baseline conditions MMPs are regulated, preventing degradation of the basement membrane. Mutations that lead to loss of control of MMPs remove the movement restriction on proliferative cells and allow mutated cells to grow beyond the basement membrane [98], [99]
7. BRCA1: BRCA1 is involved in DNA repair, entry into senescence and the expression of ER. It affects both the ability of DNA to be repaired, as well as a damaged cell’s entry into cell cycle arrest. It is complementary to p53 [100]–[102].
8. RUNX3: RUNX3 is involved in both promoting the expression of ER, as well as the suppression of c-Met. c-Met is required for proliferation; loss of suppression of c-Met allows cells to proliferate. Failure of RUNX3 is the means by which ER+ can potentially proliferate [23]–[26], [28], [103], [104].

### DNA Damage and Repair

All members of the luminal cell line include rules for the accumulation of DNA damage and the means to deal with that damage through direct DNA repair, cell cycle arrest and apoptosis. The process flow for DNA damage/repair and consequent cell fate, and the effect of mutations (resulting from DNA damage) can be seen in Figure 3. Some of the representative genes for the functional modules included in the DEABM are involved in the process, namely p53 and BRCA1. Luminal cells possess a state variable “DNA-integrity,” initially set to an arbitrary value of 1000, where each unit is assumed to represent a gene function (of which there are two copies of the gene). Each time step ( = 1 day) cells acquired a fixed amount of DNA damage in addition to a variable amount determined by a stochastic process; similarly, cells repair a fixed amount of DNA damage each day in addition to a variable amount of DNA repair (both amounts with arbitrary values but proportional to each other). If accumulated DNA damage exceeds the amount of DNA repaired during a given time step ( = 1 day) the cell’s “DNA-integrity” is lowered by the difference. If a cell’s “DNA-integrity” falls below a threshold (arbitrarily set to 97.5% integrity), the cell with intact p53 enters cell-cycle arrest, where the cell does not divide and has a decrease in the variable rate of DNA damage acquired per time step (to simulate decreased metabolic activity of arrested cells). During cell cycle arrest the cell attempts to repair accumulated DNA damage. Both p53 and BRCA1 are needed for this full capacity to manifest; a reduction in either’s gene levels through mutation lead to some degree of decreased ability to repair its DNA-integrity. Cell-cycle arrest is maintained until the cell repairs its “DNA-integrity” to above the arrest threshold (>97.5%) or damage continues to accumulate until its “DNA-integrity” falls below the apoptosis threshold of 95% (set arbitrarily). If the “DNA-integrity” falls below the apoptosis threshold, and the cell has at least one copy of each p53 and BRCA1, then the cell dies.

DNA damage present in a cell undergoing mitosis after leaving cell cycle arrest (i.e. with a “DNA-integrity <100% but >97.5%) is accrued as mutations represented with the agent variable “new-mutations,” which is set to the difference between the cell’s “DNA-integrity” variable and 1000. There is a probability that some of these mutations will affect one of the 8 focus genes/functional modules noted above, but since the large proportion of mutations would occur in genes not significant to the current focus of the DEABM, the probability of acquiring a mutation to one of the 8 focus genes is relatively low. Therefore, the likelihood that one of the focus genes would be affected is set to the number of new mutations acquired divided by 2000 (based on the modeling assumption that each cell carries 2 copies of each gene reflected in its “DNA-integrity”). As noted above, loss of copies of these 8 focus genes have defined effects on the behavior of cells.

All daughter cells that are the product of mitosis inherit the simplified genome of their parent cell. This mechanism of inheritance produces distinct lineages of heterogeneous cells with respect to genotype and behavior. Cancer, in the DEABM, emerges when a lineage of cells acquire a series of mutations that allow overgrowth and invasion.

### Definition of Cancer Outcome and Tumor Receptor Status in the DEABM

Cancer was denoted by expansion of the luminal cell population to greater than 3000 (well over 10× the normal luminal cellular population), a point demonstrated in preliminary simulations to eventually result in complete overgrowth of the model world. This behavior was consistent with enough derangement of the system to correspond to unconstrained growth. The simulation run was stopped at this point (for computational efficiency purposes), and the outcome was deemed positive for invasive breast cancer.

In terms of characterizing the ER status of a breast tumor, there are varying qualifications for what is deemed ER-positive by pathologists, ranging from 1% to 10% of nuclei [52], [54]–[57]. In addition to this variability, there are also recognized issues related to the fact that reference tissue samples generally do not reflect the entirety of the tumor mass (sample selection variance), thereby adding error to the resultant calculation, or have varying amounts of benign breast tissue present. Furthermore, since it is likely that most luminal cells are capable of expressing ER at various times, and due to these fluctuations only a fraction of cells actually show expression of ER at any time, pathology samples only reflect a “snap shot” of the tumor’s hormone receptor potential. We have attempted to integrate the ambiguous and varied information concerning ER-positivity with a plausible mechanism-based inference: we define “ER-positivity” of breast tissue based on the ER status of normal breast tissue (which we have determined as 9%). Our rationale for this selection is that a potentially estrogen-responsive tumor would be at least as responsive as normal breast tissue.

### Implementation of Hormone Receptor Status in Luminal Cells

Luminal cells can be either estrogen receptor positive (ER+) or negative (ER-). This is determined immediately following mitosis, and is governed by a stochastic process. While the exact mechanism responsible for governing whether mammary luminal cells express ER remains unknown, the base rate of ER-alpha expression has been documented to be between 4–12% of luminal cells in normal, pre-menopausal breast tissue [17]–[22]. In the DEABM there is a 10% probability that a newly formed luminal proliferative cell will express ER in the pre-menopausal state. Additionally, RUNX3 is known to suppress expression of ER, while BRCA1 activity enhances expression of ER [101], [102], as does p53 [93]–[95]. Luminal cells can either be progesterone receptor positive (PR+) or negative (PR1). In cells expressing ER, high levels of estrogen induce expression of PR, consistent with published data demonstrating that estrogen induces expression of PR in mammary epithelium [105]. Hormone receptor status was modeled as a simple *on/off* variable, and the positive and negative feedback loops governing behavior were modeled with simple algebraic equations. ER+ luminal cells secrete amphiregulin in direct proportion to the level of circulating estrogen [76], [77], [106]–[108]. Amphiregulin, a member of the epidermal growth factor family, is taken up by fibroblasts, which in turn secrete HGF, the ultimate mitogen taken up via the c-Met receptor in ER− luminal cells [75].

## Supporting Information

Supporting Materials S1 **Table of Parameter Values and Parameter Determination Process.**
(DOCX)
