# Supplementary material for: Examining the Pathogenesis of Breast Cancer Using a Novel Agent-Based Model of Mammary Ductal Epithelium Dynamics
Source: PLoS One. 2013 May 21;8(5):e64091. doi: 10.1371/journal.pone.0064091 (PMC3660364; doi:10.1371/journal.pone.0064091)
Supplement: Supporting Materials S1 — Table of Parameter Values and Parameter Determination Process. (DOCX) [file pone.0064091.s001.docx]

**Supporting Materials S1: Table of Parameter Values and Parameter Determination Process**

| **Parameter Type** | **Parameter** | **Value** | **Description** | **Fitted?** |
| --- | --- | --- | --- | --- |
| *Diffusion Rates of Secreted Mediators (Netlogo Function for Environmental Variables)* |  |  |  |  |
|  | Amphiregulin-level | 0.8 | Extracellular level of amphiregulin | No |
|  | HGF-level | 0.8 | Extracellular level of HGF | No |
|  | TGFB-level | 0.8 | Extracellular level of TGFB | No |
|  | RANK-level | 0.8 | Extracellular level of RANK | No |
| *Activity Thresholds (Agent Variable)* |  |  |  |  |
|  | Hayflick-number | 40 max | Telomer Length, limits number of cellular divisions | No |
|  | DNA-integrity | 1000 max | Counter for total amount of genetic damage, mutation rate is 1/1000 per time step | No |
|  | Bax-Level | 60 | Activates apoptosis | Yes |
|  | HGF-threshold | 50 | Amount of HGF required to trigger mitosis | Yes |
|  | TGFB-threshold | 350 | Amount of TGF-beta required to inhibit mitosis | Yes |
|  | Rank-threshold | 120 | Amount of Rank required to trigger mitosis | Yes |
|  | Senescence-Threshold | 975 | DNA-integrity levels below threshold trigger senescence in normal cells | Yes |
| *Binding/Consumption*  *(Agent-variable)* |  |  |  |  |
|  | Amph-uptake-max | 100 | Maximum amount of amphiregulin removable from the environment by a cell | No |
|  | HGF-uptake-max | 55 | Maximum amount of HGF removable from the environment by a cell | Yes |
|  | TGFB-uptake-max | 384 | Maximum amount of TGF-beta removable from the environment by a cell | Yes |
|  | Rank-uptake-max | 204 | Maximum amount of Rank removable from the environment by a cell | Yes |

This table lists key parameter values present in the DEABM. The column entitled “Fit?” denotes as to whether the parameter value was adjusted during the calibration process. As noted in the Main Text, calibration involved the iterative process of adjusting the code, running a set of simulations subsequently evaluated for face validity, and adjusting the parameters if face validity was not met. Therefore, those parameters with a “Fit?” noted as “No” had an initial value arbitrarily set and no further adjustments were made during the calibration process. Therefore, there is a hierarchy of parameter calibration built upon those parameters that were initially set and used as reference points for subsequent alterations. This hierarchy is not intended to reflect any specified biological structure (i.e. those parameters initially locked down are not meant to be more fundamental). Rather, given the emphasis on representing the relative relationships between the various variables, a relational tuning process requires that one side of the related variables be held constant. This approach also means that the absolute value of most of the variables have no intrinsic biological meaning; the sole exception to this on the listed parameters is the value of 40 for the Hayflick Number, which is derived from the literature [[91](#_ENREF_91)].

The variables are divided into two general classes: Environmental Variables, representing substances in the extracellular milieu, and Agent Variables, representing properties and components possessed by cells. The parameter values for the Environmental Variables relate to their rate of “diffusion,” i.e. the rate and concentration that they spread from on grid space to adjacent grid spaces. This is a core function of the Netlogo software system used to implement the DEABM: the value presented represents the fraction by which the Environmental Variable is reduced each time step, where the reduced value is then distributed equally into the surrounding 8 grid spaces. The absolute value of each parameters is essentially irrelevant in the DEABM; what is important is the modeling assumption that the relative distributions and diffusion rates of the represented mediators is functionally the same.

In terms of the Agent Variables, since the rules of the DEABM take the general form of logical conditional statements, the values assigned to most of these values represent thresholds that define the transition from one set of rules to another set. This rule structure is used to represent the different types of functions performed by the cellular agents. There are two subsets of Agent Variables, categorized loosely into: 1) Activity Thresholds, which denote internal cell variables that provide “switches” between different set of rule-sets/functions, and 2) Binding/Consumption variables, which represent the ability of cellular agents to decrease the level of an Environmental Variable on its resident grid space. As a matter of procedure, the signaling Activity thresholds were set first, emphasizing the relational nature between the affected rules; subsequent to this the Binding/consumption/receptor-absorption levels were modified to obtain the final version of the DEABM presented herein.

The model outputs used in the process of calibration were based on cell population dynamics. Initially the goal was to produce dynamically stable cell populations that neither became extinct or demonstrated unconstrained growth under the influence of normal menses. Once this condition had been met, the hormone levels corresponding to pregnancy were applied and luminal cell population dynamics measured for the duration of the pregnancy and shortly thereafter, with the goal of qualitatively matching the known physiological response of breast tissue expansion during pregnancy followed by additional luminal hyperplasia during lactation.

The all the simulated experiments used to generate the data presented in this manuscript were performed with the finished DEABM, which included its mutational capability, but since the intent of the hormone cycle response simulations (Figure 4) was focused on demonstrating that the DEABM could plausibly generate normal physiological behavior, circumstances where cancers developed were not considered for these baseline validation simulations.
